# InvertypeR: Bayesian inversion genotyping with Strand-seq data

**DOI:** 10.1186/s12864-021-07892-9

**Published:** 2021-07-31

**Authors:** Vincent C. T. Hanlon, Carl-Adam Mattsson, Diana C. J. Spierings, Victor Guryev, Peter M. Lansdorp

**Affiliations:** 1 Terry Fox Laboratory, British Columbia Cancer Agency, Vancouver, British Columbia V5Z 1L3 Canada; 2 European Research Institute for the Biology of Ageing, University of Groningen, University Medical Center Groningen, 9713 AV Groningen, The Netherlands; 3 Departments of Medical Genetics and Hematology, University of British Columbia, Vancouver, British Columbia V6T 1Z4 Canada

**Keywords:** Strand-seq, Inversions, Structural variation, Bayesian genotyping

## Abstract

**Background:**

Single cell Strand-seq is a unique tool for the discovery and phasing of genomic inversions. Conventional methods to discover inversions with Strand-seq data are blind to known inversion locations, limiting their statistical power for the detection of inversions smaller than 10 Kb. Moreover, the methods rely on manual inspection to separate false and true positives.

**Results:**

Here we describe “InvertypeR”, a method based on a Bayesian binomial model that genotypes inversions using fixed genomic coordinates. We validated InvertypeR by re-genotyping inversions reported for three trios by the Human Genome Structural Variation Consortium. Although 6.3% of the family inversion genotypes in the original study showed Mendelian discordance, this was reduced to 0.5% using InvertypeR. By applying InvertypeR to published inversion coordinates and predicted inversion hotspots (*n* = 3701), as well as coordinates from conventional inversion discovery, we furthermore genotyped 66 inversions not previously reported for the three trios.

**Conclusions:**

InvertypeR discovers, genotypes, and phases inversions without relying on manual inspection. For greater accessibility, results are presented as phased chromosome ideograms with inversions linked to Strand-seq data in the genome browser. InvertypeR increases the power of Strand-seq for studies on the role of inversions in phenotypic variation, genome instability, and human disease.

**Supplementary Information:**

The online version contains supplementary material available at 10.1186/s12864-021-07892-9.

## Background

The contribution of structural variants to human genetic diversity and phenotypic variation may rival that of single nucleotide variants [1]. Whereas copy number variants are readily detected using either short- or long-read human sequencing techniques and microarrays, the detection of inversions is more challenging [2]. This is especially true for inversions flanked by stretches of repetitive DNA that exceed 10 Kb. As a result, inversions are probably the most poorly-characterized structural variants in human genomes [3].

Nonetheless, inversions are known to cause phenotypic variation and disease, including microdeletion and microduplication syndromes, by suppressing recombination and disrupting genes or regulatory regions [3–7]. Methods that map inversions genome-wide will facilitate many novel studies in medical genetics including studies of their functional consequences. For such studies, heterozygous inversions should ideally be phased. This type of analysis has become possible using Strand-seq, a short read single cell sequencing method that preserves directional and haplotype information in DNA strands along entire chromosomes [8]. Strand-seq reads capture only one of the two strands of DNA for each homolog, meaning that inversions are visible as groups of mapped reads with a different orientation than their neighbours [3, 8–11].

To genotype germline inversions with Strand-seq, previous studies have relied on automated de novo discovery and manual inspection [3, 11, 12] using bioinformatic programs InvertR and BreakpointR [13]. Because these programs scan the whole genome, they may produce false positives when several adjacent reads are mis-oriented by chance, for example, due to incomplete bromodeoxyuridine incorporation [14]. This problem is typically addressed by excluding inversions with support from fewer than 50 reads [3]. Instead, we propose that focussing the analysis on published inversion coordinates and inversion-prone regions can prevent false positives while improving the detection of small inversions. Thousands of inversions are described in public databases such as in dbVAR [15], DGV [16], and invFEST [17], and we explored incorporating these coordinates into a computational genotyping step to improve inversion detection.

To this end, we present InvertypeR, a Bayesian inversion genotyping tool that works with user-supplied inversion coordinates. We validate InvertypeR using the “gold standard” inversion callset and the Strand-seq data reported by the Human Genome Structural Variation Consortium (HGSVC; [3]). The HGSVC inversion callset, produced using multiple sequencing platforms, details inversions for nine individuals (three trios) with diverse ethnic backgrounds. Here we show that InvertypeR found most inversions called by the HGSVC but also generated multiple credible phased inversion calls that were not previously reported.

## Results

**Fig. 1:**
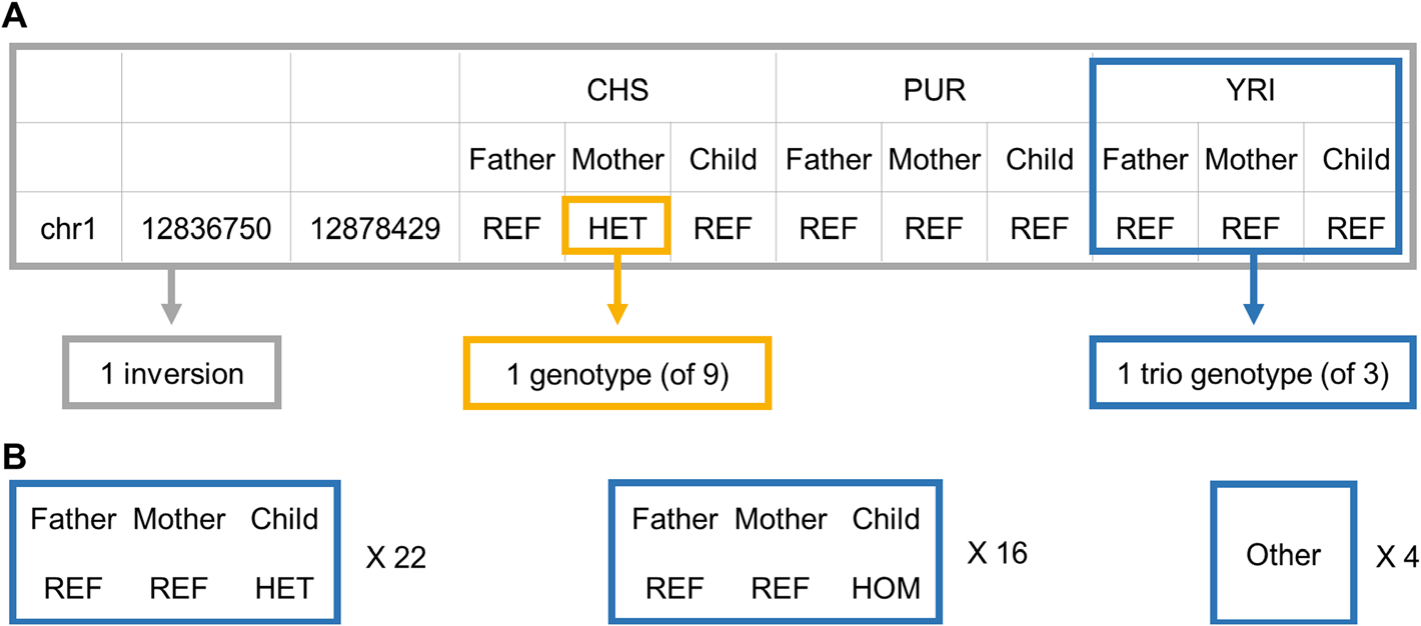
**A** A visual glossary: We use the term “inversion” broadly for general properties such as coordinates and size, or collectively for all genotypes. We use the term “genotype” for the inversion state (REF, HET, HOM, or ambiguous) in one of the 9 individuals. “Trio genotype” refers to a complete set of three non-ambiguous genotypes for a trio, which can then be checked for Mendelian concordance. **B** Summary of the 42 instances of Mendelian discordance among the HGSVC trio genotypes

**Fig. 2:**
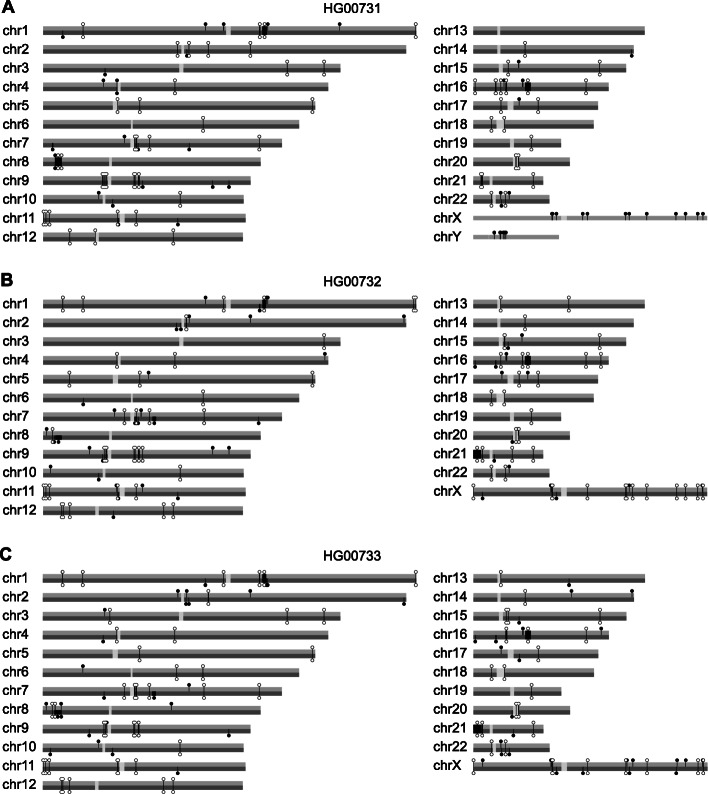
Output of InvertypeR. The locations of HOM (open lollipops) and phased HET inversions (closed lollipops) are shown for each individual of one of the HGSVC trios (of Puerto Rican descent; see [3]): **A** HG00731, **B** HG00732, and **C** HG00733. In the related Supplemental Figs. (S2-S10, Additional files 2, 3, 4, 5, 6, 7, 8, 9 and 10), clicking any lollipop links to Strand-seq data and other pertinent features displayed in the UCSC Genome Browser [20]. The sizes of inversions larger than 50 Kb are indicated with black bars. Inversion coordinates are from a combination of the de novo and public callsets (below)

We noticed that 6.3% (42/667) of the inversion genotypes reported for the three HGSVC trios [3] showed Mendelian discordance, in that a parent did not have one of the child’s two alleles (Fig. 1, Supplemental Figure S1, Additional file 1). This is suggestive of genotyping errors because de novo large inversions are thought to arise very infrequently (10^− 5^–10^− 4^/generation [18, 19]). We reasoned that two innovations might enable more accurate inversion genotyping. First, using fixed genomic inversion coordinates to analyze Strand-seq data could increase statistical power and allow smaller inversions to be genotyped. Second, using a novel Watson-Crick (“WC”) composite Strand-seq file could distinguish heterozygous inversions (“HET”) from alignment errors, reference assembly collapses, and homozygous inversions (“HOM”; see Methods), while also phasing HET inversions. For this purpose, we developed a Bayesian bioinformatic program in R, InvertypeR, that analyses Strand-seq data to generate genome-wide phased inversion genotypes. Using this program, all inversions are plotted onto chromosome ideograms (Fig. 2; Additional files 2, 3, 4, 5, 6, 7, 8, 9 and 10) with direct links for each phased inversion to Strand-seq data displayed in the UCSC Genome Browser [20].

We validated InvertypeR using the Strand-seq data and 2043 inversion genotypes published by the HGSVC ([3]; Supplemental Results, Additional file 1). InvertypeR found confident genotypes for 74.7% of these 2043 HGSVC genotypes, making ambiguous calls for the remaining 25.3%, where an ambiguous call means that no genotype had a posterior probability greater than 95% (Table 1; Supplemental Table S1, Additional file 1). Most (86.6%) of the confident InvertypeR genotypes matched the HGSVC genotypes, while 12.5% did not match and the remaining 0.9% were ambiguous according to the HGSVC. For more than half of the mismatches, InvertypeR called a reference homozygote (“REF”) where the HGSVC called HET (Supplemental Figure S11, Additional file 1).

**Table 1:** Comparison of HGSVC (H.) and InvertypeR (I.) inversion genotype calls obtained with the same Strand-seq data (9 individuals × 227 simple inversions = 2043 genotypes). The HGSVC also used multiple additional data types (e.g., PacBio)

Inversion genotypes				
2043				
H. and I. confident genotypes		H. or I. ambiguous genotypes		
1512		531		
H. and I. agree	H. and I. disagree	I. ambiguous	H. ambiguous	Both ambiguous
1321	191	516	14	1

Inversion genotypes obtained by InvertypeR showed less Mendelian discordance than those reported by the HGSVC. The InvertypeR genotypes corresponded to 432 “trio genotypes”, that is, where confident genotypes were reported for all three individuals of a trio at a given inversion (Fig. 1a). Of these, two (0.5%) showed Mendelian discordance. By contrast, for the corresponding 425 trio genotypes in the HGSVC callset, 14 (3.3%) showed Mendelian discordance; this figure rose to 17.7% if we considered only the 79 trio genotypes where InvertypeR and the HGSVC differed.

**Fig. 3:**
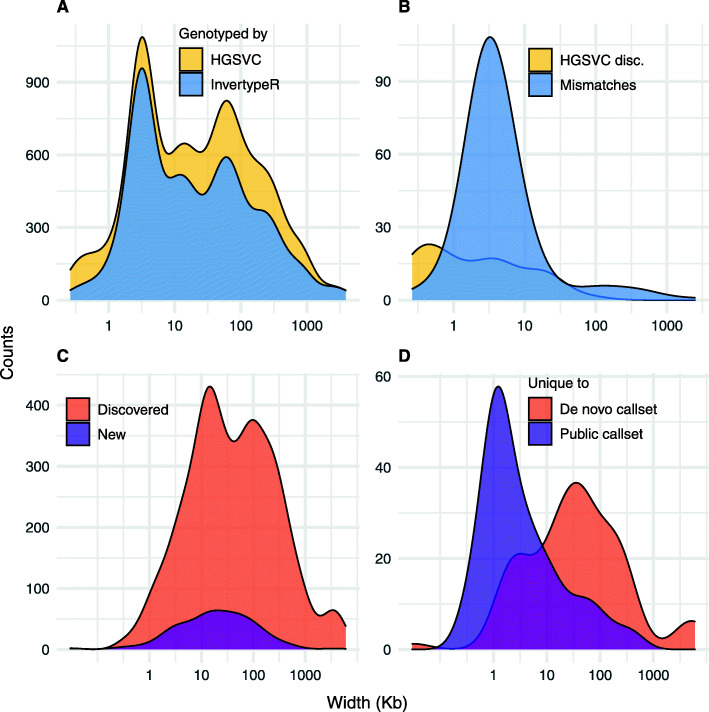
Size distributions of inversions for **A** all 2043 genotypes called by the HGSVC (yellow) and those which were re-genotyped in this study (light blue), **B** the HGSVC trio genotypes that show Mendelian discordance (yellow) and all trio genotypes with at least one mismatch between the InvertypeR and HGSVC genotypes (light blue), **C** all inversions discovered using the de novo callset or the public callset (red) and new inversions not reported by the HGSVC (purple), and **D** the inversions unique to the de novo callset (red) and those unique to the public callset (purple)

InvertypeR was able to genotype inversions in the size range 263 bp – 3.9 Mb (Fig. 3a). Notably, InvertypeR’s ambiguous calls (517 of 2043 genotypes) were generally at large inversions (median 41.1 Kb vs. 18.4 Kb for all inversions). However, InvertypeR also missed very small inversions < 1 Kb (Fig. 3a), presumably reflecting sparse Strand-seq data. Mismatches between the InvertypeR and HGSVC genotypes were more common among small inversions (median size 3.2 Kb), as were inversions showing Mendelian discordance for the HGSVC genotypes (median 1.5 Kb; Fig. 3b). We conclude that InvertypeR primarily improves the genotyping of small inversions.

InvertypeR can be run on arbitrary inversion coordinates submitted by the user. We therefore used two additional sources of coordinates to discover inversions beyond the HGSVC callset (Additional files 1 & 11). First, we compiled an inversion catalogue of 8052 published inversions and 735 inversion-prone Terminal Inverted Repeat spacers (“TIRs” [21]), which we genotyped with InvertypeR (“public callset”). Second, we ran BreakpointR [13] on the composite files to discover switches in strand state, which we genotyped with InvertypeR (“de novo callset”). We found 66 inversions not reported by the HGSVC (median size 25.0 Kb; Fig. 3c-d), of which 25 were novel, that is, they were not among the 8052 published events in the inversion catalogue. Of these, six were inverted TIRs unique to the public callset and nine were on the Y chromosome, which was excluded by the HGSVC [3]. Moreover, when we re-genotyped all inversions in the de novo and public callsets (both novel and previously-described) at a standard set of coordinates generated for each trio (Supplemental Results, Additional file 1), the vast majority showed Mendelian concordance (98.9%).

## Discussion

InvertypeR generates accurate and reproducible phased inversion genotypes from Strand-seq data at user-specified coordinates. Previously, inversion calls relied on the manual inspection of strand switches identified by InvertR or BreakpointR [3, 11]. InvertypeR replaces this qualitative process with a quantitative Bayesian model to associate genotypes with posterior probabilities, which reduces the scope for user errors and allows rigorous comparisons between inversion callsets. Moreover, while BreakpointR coordinates can still be used as input, using known inversion coordinates improves the detection of inversions smaller than 10 Kb.

**Fig. 4:**
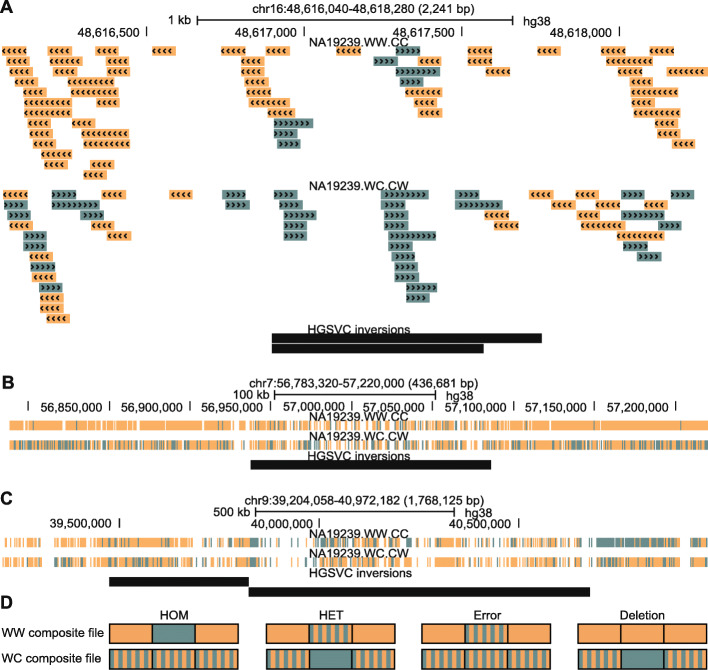
Genomic tracks showing evidence for genotype calls from Strand-seq composite files (forward reads green, reverse reads orange) at HGSVC inversion coordinates (black bars; [3]). **A** This inversion is HET according to both InvertypeR and manual inspection but is REF according to the HGSVC. **B** InvertypeR called this ambiguous, the HGSVC called HET, and manual inspection shows this to be an alignment error or reference assembly collapse because the WC composite file (bottom) has no strand switch. The HGSVC [3] did not use a WC composite file and may have missed this. **C** Two adjacent HET inversions according to the HGSVC. InvertypeR returns an ambiguous call. Manual inspection indicates unclear strand-switches and likely alignment errors. **D** A guide to manual inspection. The second box in each triple represents the putative inversion, while the first and third represent flanking sequences. Putative inversions are likely false positives unless the strand states clearly match the patterns shown for HOM or HET. Note that the colour of the second box for deletions and HET inversions may be either solid orange or solid green (i.e., just not WC)

In 17.7% of cases where InvertypeR returned different genotypes for a trio than those reported previously [3], the published genotypes showed Mendelian discordance. This is strong evidence that many more than 17.7% of these mismatches are due to errors in the HGSVC callset, since most single-allele genotyping errors do not affect concordance (e.g., father/mother/child REF/REF/REF vs. HET/REF/REF). Such errors may reflect the difficulty of reconciling conflicting genotypes from the multiple technologies used by the HGSVC. InvertypeR corrected the Mendelian discordance for a third (14/42) of discordant trio genotypes and returned at least one ambiguous call for the rest (28/42), indicating that the underlying Strand-seq data was of poor quality. Furthermore, manual inspection of the Strand-seq data at HGSVC-InvertypeR mismatches also supports the genotypes inferred by InvertypeR (e.g., Fig. 4a).

The HGSVC enlisted five techniques in addition to Strand-seq to call and genotype inversions [3]. Using the Strand-seq data alone, InvertypeR could unambiguously re-genotype the majority of them (74.7%). Surprisingly, for 83.0% (429/517) of InvertypeR’s ambiguous calls, the corresponding HGSVC genotypes were called using Strand-seq data [3]. But inversions where the HGSVC called HET for the vast majority of individuals (at least seven of the nine) account for 42.7% of these 429 ambiguous calls, that is, at least 4.75-fold more than expected (Supplemental Results, Additional file 1). Such inversions are most likely caused by collapses in the reference assembly, which appear as fixed HET inversions in the Watson-Watson (“WW”) Strand-seq composite files used by the HGSVC. InvertypeR can distinguish HET inversions from assembly collapses because the latter do not show a strand switch in our novel WC composite files (Fig. 4a-b; see Methods), and in such cases it returns an ambiguous call to reduce the false positive rate.

For all 517 of InvertypeR’s ambiguous genotype calls, two further lines of evidence suggest that the Strand-seq data truly do not support a genotype call. First, nearly four-fold more of the HGSVC trio genotypes show Mendelian discordance where InvertypeR found at least one ambiguous call (11.6%) than where InvertypeR found no ambiguous calls (3.3%). Second, manual inspection of the Strand-seq data shows that InvertypeR’s ambiguous calls often have no support from the WC composite file or unclear strand switches (e.g., Fig. 4b-c).

We genotyped putative inversion coordinates obtained from two complementary callsets to find 29.1% (66/227) more inversions than were reported in the HGSVC gold standard. Using 8787 published inversion coordinates and TIR spacers, InvertypeR found inversions smaller than 10 Kb that were missed by BreakpointR because of its lower statistical power (Fig. 3d; [13]). For discovering novel inversions or when published coordinates are incorrect, however, InvertypeR can use coordinates generated by BreakpointR to resolve medium and large inversions that cannot otherwise be genotyped. We recommend using both the published coordinates and de novo BreakpointR coordinates with InvertypeR for comprehensive discovery and genotyping of inversions with Strand-seq data.

A limitation of InvertypeR is that it is not well-suited to the characterization of complex inversions, such as inverted duplications, since it does not attempt to infer copy number. It may identify whether an inverted copy is present, but in 82.1% of cases it returns an ambiguous call (Supplemental Table S2, Additional file 1). Similarly, due to a bin size requirement, composite files may not render extremely large inversions correctly (> 8 Mb for HET, > 4 Mb for HOM), making them difficult to genotype. Fortunately, the genotypes of such large events are readily evident in the chromosome ideograms plotted by BreakpointR [13].

## Conclusions

As more inversions are discovered and linked to phenotypes, genotyping known coordinates may become more pressing than the search for rare or novel inversions. Because InvertypeR can genotype an inversion with as few as 10 reads without the necessity of confirmation from other techniques, low-coverage Strand-seq data could be used to genotype medically-relevant inversions at comparatively low cost (57.6% of inversions can be genotyped with just 5% reads; Supplemental Table S3, Additional file 1). Combined across hundreds of individuals, such data could also provide estimates of population-level allele frequencies. InvertypeR provides a reproducible, quantitative framework for inversion genotyping that makes the complex genetic information present in Strand-seq data more accessible to the wider genomics community.

## Methods

### Composite BAM files

WW composite BAM files were created by combining WW and CC regions identified with BreakpointR (i.e., regions where Strand-seq reads are nearly all oriented in the same direction; [13]), with reads in the former reversed by manipulating SAM flags [11]. To create WC composite files, we identified WC or CW regions (i.e., regions where Strand-seq reads are oriented in both directions) using 20 Mb bins in BreakpointR, called SNPs with freebayes [22], using generous parameters to identify putatively heterozygous SNPs in sparse Strand-seq data (Supplemental Methods, Additional file 1), and phased libraries using StrandPhaseR [9]. This allowed us to distinguish WC regions from CW regions, reverse reads in the latter by altering SAM flags, and merge reads from all libraries while maintaining directionality (Supplemental Methods, Additional file 1).

### InvertypeR

First, to avoid bias from rare inversions and poorly-mapped regions, we estimate the background (the proportion of non-directional reads) by making a kernel density estimate of the mode background of 1 Mb genomic bins. We use reads with mapping quality of at least 10 that do not intersect a blacklist (Supplemental Methods, Additional file 1). Given counts of Watson (“W”) and Crick (“C”) reads within a putative inversion, either the background or 0.5 can be used as the success probability *θ* in a binomial distribution to compute the likelihoods of the four possible strand states - WW, WC, CW, and CC - in each composite file (denoted ${L}_{strand\ state}^{composite\ file}={L}_{WW}^{WC}$, etc.). If we assume that there are four possible genotypes, REF, HET (0|1), HET (1|0), and HOM, with prior probabilities *P*_*REF*_, *P*_*HET*_, etc., and that the underlying error states of the Strand-seq data can be described exclusively as “no error”, “always WC or CW”, or “missing/deletion” (Supplemental Table S4, Additional file 1), then we can write the likelihoods of the genotypes. For example:

$$ {L}_{HOM}={P}_{HOM}\left[\frac{1}{2}{P}_{none}{L}_{CC}^{WW}\ {L}_{WC}^{WC}+{P}_{AWC}{L}_{WC}^{WW}\ {L}_{WC}^{WC}+\frac{1}{2}{P}_{del}{L}_{CC}^{WW}\left({L}_{WW}^{WC}+{L}_{CC}^{WC}\right)\right] $$

and,

$$ {L}_{HET\left(0|1\right)}={P}_{HET}\left[{P}_{none}{L}_{WC}^{WW}\ {L}_{WW}^{WC}+{P}_{AWC}{L}_{WC}^{\mathrm{W}W}\ {L}_{WC}^{WC}+\frac{1}{2}{P}_{del}\left({L}_{WW}^{WW}+{L}_{CC}^{WW}\right){L}_{WW}^{WC}\right], $$

where *P*_*none*_, *P*_*AWC*_, and *P*_*del*_ represent prior probabilities associated with each of the three error states, for which we chose conservative values of 0.5, 0.25, and 0.25, respectively. We used log-likelihood versions of these equations to improve the accuracy of computation and applied Bayes Theorem to find the genotype with the largest posterior probability. We considered any genotype call with a posterior probability of at least 95% to be a confident call.

We addressed the problem of inaccurate or overlapping inversion coordinates by providing two optional start- and end-point adjustment routines, followed by re-genotyping. For confident non-REF inversions, the user can merge overlapping inversions of the same genotype. Alternatively, for either confident non-REF inversions or for ambiguous inversions, InvertypeR can move start- and end-points to nearby peaks in deltaW values summed over a range of bin sizes [13], which approximate the greatest change in read directionality. We retain adjusted inversion coordinates if InvertypeR returns a confident genotype that matches the original genotype (if applicable).

### Inversion catalogue

We compiled an extensive catalogue of 8787 previously characterized inversions and TIRs, using coordinates described in dbVAR [15], DGV [16], and invFEST [17], together with those reported by [3] and [11]. We also included 735 TIRs, since segmental duplications often flank inversions. When counting 80% overlapping intervals only once, the 8787 events of the inversion catalogue correspond to roughly 3701 unique intervals (Supplemental Methods, Additional file 1).

### Validation: HGSVC trios

We used three different sets of Bayesian priors, corresponding to three different sets of putative inversion coordinates, to genotype the HGSVC trios. For the 227 simple inversions [3], we used the reported frequency of each genotype as the prior for that individual (e.g., HG00732 has 101 REF and 1 ambiguous call, so ${P}_{REF}=\frac{101}{227-1}=0.4469$). For the public callset, we used

$$ {P}_{HET}={P}_{HOM}=\frac{ni}{2{l}^2} $$

$$ {P}_{RE F}={P}_{RE{F}_{male}}=1-{P}_{HOM}-{P}_{HET} $$

where *n* is the number of expected inversions, *i* is the number of self-intersections of the inversion catalogue (requiring 80% reciprocal overlap, i.e., bedtools intersect -f 0.8 -r -a inversions -b inversions [23]), and *l* is the total number of inversions in the catalogue. We set *n* = 100 based on experience with test data. The equation above represents the expected frequency of biological inversions for a list of putative inversion coordinates, using $\frac{l^2}{i}$ to estimate the number of unique putative inversions if there are overlaps. For the de novo callset, we repeated the calculation using the BreakpointR intervals (below) for *i* and *l*, and we chose *n* = 80 because we predicted that smaller inversions would be missed.

We ran BreakpointR [13] three times on the composite files to capture novel inversions of different sizes and that could not be genotyped using published coordinates. We set binMethod = “reads” and used windowsize = 40, 120, and 360 reads, with minReads = 15, 50, and 50 reads, respectively. We then genotyped all intervals from the three BreakpointR runs and both composite files for which the strand state did not match the base state (Supplemental Methods, Additional file 1). We used the UCSC Genome Browser to visualize some inversions [20].

## Supplementary Information

**Additional file 1.** Supplemental Results, Supplemental Methods, Supplemental Tables, and Supplemental Figures.

**Additional file 2: Supplemental Figure S2.**

**Additional file 3: Supplemental Figure S3.**

**Additional file 4: Supplemental Figure S4.**

**Additional file 5: Supplemental Figure S5.**

**Additional file 6: Supplemental Figure S6.**.

**Additional file 7: Supplemental Figure S7.**

**Additional file 8: Supplemental Figure S8.**

**Additional file 9: Supplemental Figure S9.**

**Additional file 10: Supplemental Figure S10.**

**Additional file 11.** Supplemental Data showing raw inversion calls, blacklisted regions, and the inversion catalogue for the public callset.

## Data Availability

The inversions and genotypes described in this study are available under accession number nstd198 in the NCBI dbVar database, https://www.ncbi.nlm.nih.gov/dbvar/studies/nstd198/. InvertypeR is available as an R package on GitHub (https://github.com/vincent-hanlon/InvertypeR), along with a step-by-step guide to the analysis and the scripts used to generate composite files and plot inversions. The Strand-seq data [3] analysed during this study are available from the International Genome Sample Resource, https://www.internationalgenome.org/data-portal/data-collection/structural-variation. The inversion calls in [3] that we re-analyzed are available as Supplementary Data 9 & 43 of that study (10.1038/s41467-018-08148-z).

## Abbreviations

C: Crick
CC: Crick-Crick
HET: Heterozygous
HGSVC: Human Genome Structural Variation Consortium
HOM: Homozygous alternate
REF: Homozygous reference
TIRs: Terminal inverted repeat spacers
W: Watson
WC: Watson-Crick
WW: Watson-Watson
