# Supplementary material for: InvertypeR: Bayesian inversion genotyping with Strand-seq data
Source: BMC Genomics. 2021 Jul 31;22:582. doi: 10.1186/s12864-021-07892-9 (PMC8325862; doi:10.1186/s12864-021-07892-9)
Supplement: Supplementary file 1 — Additional file 1. Supplemental Results, Supplemental Methods, Supplemental Tables, and Supplemental Figures. [file 12864_2021_7892_MOESM1_ESM.docx]

# Additional file 1

**Table of Contents**

[Supplemental Results 1](#_Toc57017940)

[Supplemental Methods 4](#_Toc57017941)

[Supplemental Tables 7](#_Toc57017942)

[Supplemental Figures 11](#_Toc57017943)

[References 16](#_Toc57017944)

# **Supplemental Results**

Validation of InvertypeR

The HGSVC provided two sets of genomic coordinates for their 227 simple inversions: an outer (wider) set and an inner (narrower) set. We ran InvertypeR on both sets of coordinates (using the setting adjust_method=”low”). Where InvertypeR produced confident genotypes (i.e., with a posterior probability of at least 95%) at both the inner and the outer coordinates for an inversion in an individual, the two genotypes were identical in all but ten cases (at eight inversion loci). For these eight inversions, we report the genotypes of the inner coordinates for all nine individuals, reasoning that if the outer coordinates are wider than the inversion itself, they may produce an erroneous genotype by including non-inverted reads nearby (e.g., Supplemental Figure S12). In all ten cases, the genotypes of the outer coordinates were reference homozygote (“REF”) while the genotypes of the inner coordinates were heterozygote (“HET”) or alternate homozygote (“HOM”). When reporting the sizes of inversions, we used the outer coordinates rather than the inner coordinates.

During genotyping for HG00731, InvertypeR merged two adjacent HOM inversions and reported them as one event. For the comparison with the HGSVC genotypes we used the HGSVC inversion coordinates, and we used InvertypeR’s HOM genotype for both (unmerged) inversions. We did the same when InvertypeR merged two adjacent HOM inversions during the downsampling experiment (Supplemental Table S3).

Manual inspection shows that the two InvertypeR trio genotypes with Mendelian discordance (see Results) both result from inversions that were incorrectly called REF because of excessively wide inversion coordinates (above; e.g. the outer coordinates in Supplemental Figure S12).

Inversion discovery with InvertypeR

We found 1007 and 870 confident non-REF inversion genotypes using the public callset and the *de novo* callset, respectively (median size 28.2 Mb and 46.7 Mb), for a combined total of 230 inversions when overlapping events were merged across all individuals and both callsets. Of these, 42 were unique to the public callset and 30 were unique to the *de novo* callset. Inversions discovered exclusively using the public callset were generally smaller than those unique to the *de novo* callset (median size 1.7 Kb vs 36.6 Kb; Figure 3). 66 inversions from the two callsets were not among the simple inversions, the complex inversions, or the misoriented reference sequences reported by the HGSVC. Of these 66 new inversions, 18 were unique to the public callset and 22 were unique to the *de novo* callset. 17 of the 66 inversions were on the Y chromosome, which was excluded from the HGSVC study. Interestingly, 15 of these were polymorphic among males, confirming that they were not misoriented reference sequences. InvertypeR alone cannot distinguish misoriented reference sequences from HOM inversions, however, and it is also possible that some of the autosomal calls in the *de novo* and public callsets are not biological inversions.

Using InvertypeR to genotype coordinates generated by BreakpointR (1) is well-suited to the discovery of novel inversions. We found 14 novel inversions unique to the *de novo* callset (i.e., they did not overlap the inversion catalogue, TIRs excluded, using bedtools intersect -v -f 0.1 -r). Altogether, after accounting for overlapping events, combining the two callsets yielded 25 novel inversions with a median unmerged size of 29.6 Kb (using *de novo* callset coordinates for inversions in both callsets).

By either merging or intersecting overlapping inversions found in members of the same trio, we produced two standard sets of inversion coordinates for further comparison. After genotyping these at fixed coordinates and with the same Bayesian prior as before, we examined inversions for which InvertypeR produced confident trio genotypes at both the merged and the intersected coordinates, and for which the two trio genotypes were identical. When the REF prior probability was greater than our 95% confidence threshold, we excluded REF genotypes for which the calculated posterior was within 0.1% of the prior. Such genotypes are unreliable because they indicate that no information about the inversion is present in the Strand-seq data.

We then checked the trio genotypes that met these criteria for Mendelian discordance. For each trio, we combined the two callsets by taking all of the *de novo* callset trio genotypes and adding in any public callset trio genotypes the did not intersect the *de novo* callset. This left 263 trio genotypes, of which all but three showed Mendelian concordance.

Expected frequency of majority-HET inversions

Many of the genotypes that InvertypeR called ambiguous (but for which the HGSVC reported support from Strand-seq data) were at inversions where the HGSVC called HET for at least seven of the nine individuals (see Discussion). We can estimate the maximum expected frequency of polymorphic inversions for which at least seven of the nine individuals are HET by assuming allele frequencies $p=q=0.5$ for the inverted and non-inverted alleles (other values of $p$ and $q$ will give smaller estimates). Then, the expected frequency of HET genotypes is $2pq=0.5$, and the frequency of inversions for which at least seven of nine individuals are HET is given by the binomial distribution:

$$\sum_{x=7}^{9} \binom{9}{x}\left( 0.5 \right)^{x}{(1-0.5)}^{9-x}=8.98\%$$

# **Supplemental Methods**

Blacklist construction

The blacklist was constructed by fitting a normal distribution to the natural logarithm of 10 Kb binned read counts for all nine individuals from (2) to identify bins outside the three-sigma limits (Supplemental Data 23, Additional file 11). We first fit a normal distribution to the autosomal bins, with a kernel density estimate of the mode used as the mean. To avoid bias from empty bins, we used only bins larger than the mode to estimate the standard deviation. We excluded autosomal bins with a read count greater than $b=e^{\mu+3\sigma}$. For the sex chromosomes, we transformed $b$ to account for read depth by counting the total number of mapped reads for male samples ($m$) and for female samples ($f$). For the X chromosome, we excluded bins with a read count greater than

$$b_{X}=b\frac{f+\frac{1}{2}m}{m+f}$$

For the Y chromosome, we excluded bins with a read count greater than

$$b_{Y}=b\frac{\frac{1}{2}m}{m+f}$$

We also excluded modelled centromeric sequences (3).

Composite files

All composite files were created using the blacklist to mask error-prone regions when identifying WW, WC, and CC regions with BreakpointR (1). A 3.7 Mb inversion interfered with the representation of the Y chromosome in the WW composite file for NA19239, by preventing BreakpointR from identifying WW and CC regions with the window size we used. We addressed this anomaly by masking the inversion during the BreakpointR step only (the inversion is genotyped by InvertypeR regardless).

For WC composite file creation, we ran freebayes (-n 2 -C 1 -K -F --haplotype-length 0) to identify all possible biallelic SNPs based on very few reads (4). We selected SNPs where the alternate allele was supported by at least two reads and the reference allele was supported by at least one read, and we used these to distinguish WC and CW regions with StrandPhaseR (5).

We validated all composite files using GATK’s ValidateSamFile and gunzip -t.

The public and *de novo* callsets

We used both published inversion coordinates (public callset) and *de novo* strand switches (*de novo* callset) to discover inversions beyond the HGSVC callset with InvertypeR. While genotyping for the public callset, we used InvertypeR to adjust inversion start- and end-points both for confident non-REF calls and for ambiguous calls (adjust_method=”all”; Methods). While genotyping for the *de novo* callset, we used InvertypeR to adjust inversions for confident non-REF inversions only (adjust_method=”deltas”; Methods). This allowed us to combine overlapping BreakpointR strand switches from the two composite files (1). When genotyping standardized inversion coordinates to verify Mendelian concordance (Supplemental Results), we did not adjust inversion start- or end-points using InvertypeR (adjust_method=”raw”).

We combined inversions from the two callsets and removed inversions from the public callset that overlapped inversions in the *de novo* callset to plot the chromosomal ideograms (Figure 2 and Supplemental Figures S2-S10). Because InvertypeR adjusts inversion coordinates based on read position, inversions adjacent to centromeres or telomeres sometimes incorrectly span large read-poor regions (e.g., HG00512 chr9; see Supplemental Figure S2). InvertypeR uses read density to identify such inversions, and we recommend checking their coordinates by manual inspection using a genome browser.

Inversion catalogue

We collected accession numbers for each inversion along with GENCODE (V32) gene symbol(s) for those that overlapped one or more genes (Supplemental Data 24, Additional file 11). For inversions described in invFest, we also included metadata related to validation, global frequency and functional effects. No downstream processing or filtering (e.g., merging overlapping events) was applied to the inversion catalogue. Inversion coordinates are for reference genome build GRCh38, and coordinates originally given for other reference builds were lifted to GRCh38 using LiftOver (https://genome.ucsc.edu/cgi-bin/hgLiftOver). For the public callset (see Results), we included a shortened version of each inversion as well: for coordinates $(chr, start, end)$ we included $(chr, start+length/4, end-length/4)$.

# **Supplemental Tables**

**Supplemental Table S1.** Comparison of InvertypeR and HGSVC genotype calls by trio. The trio CHS is from the Han Chinese South population (HG00512, HG00513, HG00515), the trio PUR is from the Puerto Rican population (HG00731, HG00732, HG00733), and the trio YRI is from the Yoruba population in Ibadan, Nigeria (NA19239, NA19238, NA19240). Ambiguous calls are written “NA”, and the HGSVC’s deletion calls are written “del”.

| **Genotype call** | | **# Genotypes** | | | |
| --- | --- | --- | --- | --- | --- |
| **HGSVC** | **InvertypeR** | **All** | **CHS** | **PUR** | **YRI** |
| HET | HET | 261 | 83 | 83 | 95 |
| HET | HOM | 34 | 13 | 12 | 9 |
| HET | NA | 322 | 105 | 112 | 105 |
| HET | REF | 112 | 37 | 35 | 40 |
| HOM | HET | 1 | 0 | 0 | 1 |
| HOM | HOM | 276 | 93 | 85 | 98 |
| HOM | NA | 48 | 13 | 19 | 16 |
| HOM | REF | 16 | 6 | 7 | 3 |
| NA | HOM | 3 | 2 | 0 | 1 |
| NA | NA | 1 | 0 | 0 | 1 |
| NA | REF | 11 | 3 | 4 | 4 |
| REF | HET | 10 | 2 | 4 | 4 |
| REF | HOM | 17 | 8 | 5 | 4 |
| REF | NA | 144 | 50 | 49 | 45 |
| REF | REF | 784 | 263 | 266 | 255 |
| del | HOM | 1 | 1 | 0 | 0 |
| del | NA | 2 | 2 | 0 | 0 |

**Supplemental Table S2.** Comparison of HGSVC and InvertypeR genotypes for the HGSVC’s complex inversions, that is, inversions with copy number changes. InvertypeR generally returns an ambiguous call (“NA”) in such cases. We used prior $P_{REF}=0.1$, and we set $P_{HET}$ and $P_{HOM}$ according to the frequency of those genotypes in the HGSVC results. We also used the setting adjust_method=”low”.

| **HGSVC** | **InvertypeR** | **# Genotypes** |
| --- | --- | --- |
| HET | HET | 8 |
| HET | HOM | 2 |
| HET | NA | 243 |
| HET | REF | 31 |
| HOM | HET | 1 |
| HOM | HOM | 8 |
| HOM | NA | 13 |
| HOM | REF | 6 |
| NA | NA | 5 |
| NA | REF | 1 |

**Supplemental Table S3.** InvertypeR genotypes with a simulated low-coverage BAM file (to mimic a shallow sequencing run). Full: using InvertypeR on the original BAM files for HG00512 to genotype the HGSVC’s 227 simple inversions (total 172 confident genotypes). Downsampled: using InvertypeR on the BAM files after 95% of reads have been randomly removed (total 99 confident genotypes; samtools -F1024 -bs117.05). Ambiguous calls are written “NA”.

| **Full** | **Downsampled** | **# Genotypes** |
| --- | --- | --- |
| REF | REF | 61 |
| REF | NA | 48 |
| HET | REF | 2 |
| HET | HET | 11 |
| HET | NA | 11 |
| NA | REF | 6 |
| HOM | REF | 2 |
| HOM | HOM | 23 |
| HOM | NA | 14 |
| NA | HOM | 3 |
| NA | NA | 46 |

**Supplemental Table S4.** Interpretation of strand states in a composite file. Reference assembly collapses and alignment errors appear as AWC (Always Watson-Crick), that is, with both forward and reverse reads in each composite file. This allows them to be distinguished from HET inversions. Similarly, neither composite file has both forward and reverse reads at a locus with a heterozygous deletion.

| **Strand state** | |  |  |
| --- | --- | --- | --- |
| **WC composite file** | **WW composite file** | **Inversion state** | **Error state** |
| WC | WW | REF | none |
| WC | CC | HOM | none |
| WW or CC | WC | HET | none |
| WC | WC | any | AWC |
| W(W) or C(C) | W(W) or C(C) | any | deletion |

# **Supplemental Figures**


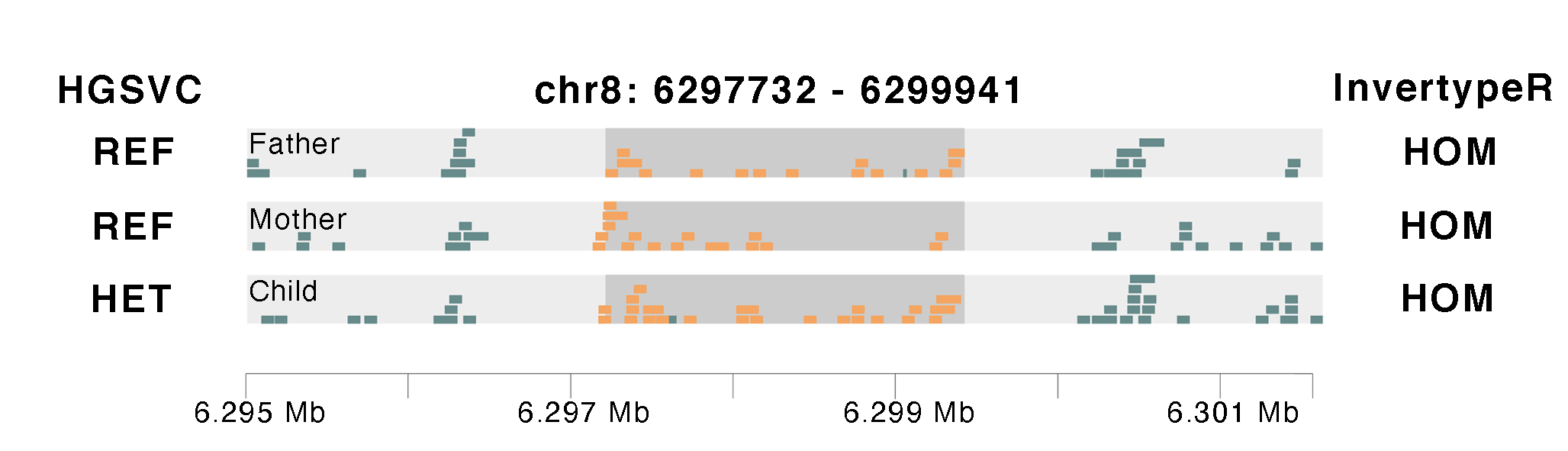


**Supplemental Figure S1.** Example of Mendelian discordance. InvertypeR and manual inspection of the Strand-seq data (from WW composite files) show a concordant trio genotype (father/mother/child HOM/HOM/HOM). The HGSVC trio genotype is discordant because an inverted allele in the child (HET) is found in neither of the parents (REF). Inversion coordinates from InvertypeR.

**Supplemental Figures S2-S10.** InvertypeR output for all 9 HGSVC individuals, using all coordinates from the *de novo* callset as well as those in the public callset which did not intersect inversions in the *de novo* callset. Open lollipops are HOM inversions, closed lollipops are phased HET inversions, and black bars show inversion size for events larger than 50 Kb. Each lollipop is a link to Strand-seq data displayed in the UCSC Genome Browser (6). In these plots, the coordinates of inversions flagged as having low read density were not adjusted manually, as recommended (Supplemental Methods). S2: HG00512. S3: HG00513. S4: HG00514. S5: HG00731. S6: HG00732. S7: HG00733. S8: NA19239. S9: NA19238. S10: NA19240. See corresponding Additional files 2-10.


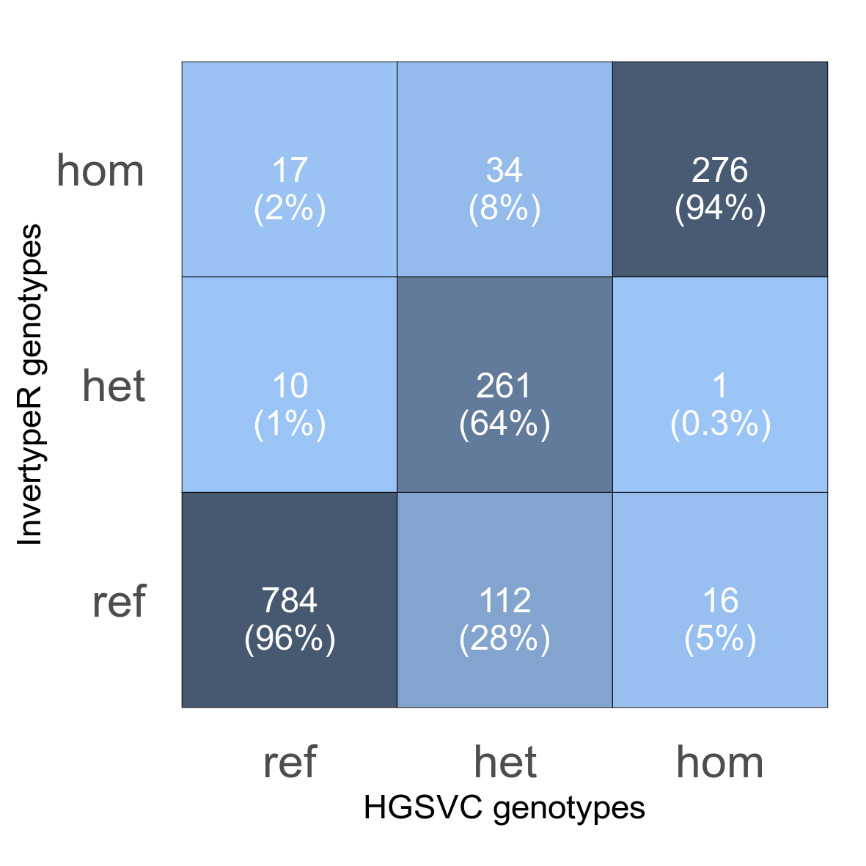


**Supplemental Figure S11.** Comparison of non-ambiguous genotypes called by both the HGSVC and InvertypeR. Most mismatches occurred where the HGSVC called HET and InvertypeR called REF. Matching genotypes are displayed along the main diagonal.

**
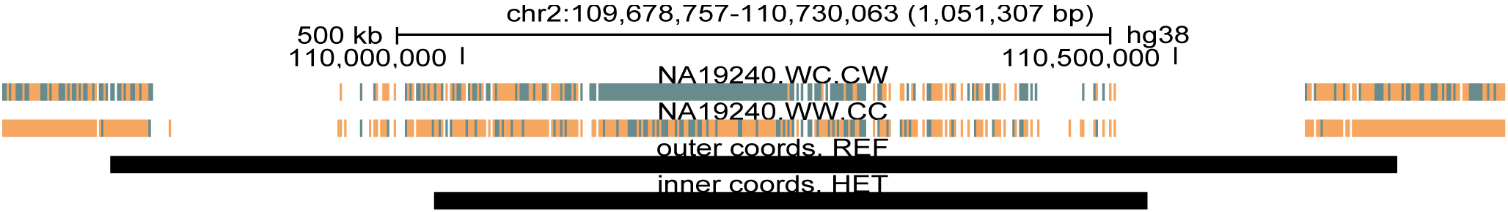
**

**Supplemental Figure S12.** Example of an inversion for which the two sets of HGSVC coordinates (inner and outer) result in conflicting genotype calls. The outer coordinates include enough non-inverted reads that InvertypeR calls REF, while the inner coordinates are accurate enough that InvertypeR can call HET. See Supplemental Results.

#

# **References**

1. Porubsky D, Sanders AD, Taudt A, Colome-Tatche M, Lansdorp PM, Guryev V. breakpointR: an R/Bioconductor package to localize strand state changes in Strand-seq data. Bioinformatics. 2020;36(4):1260-1.

2. Chaisson MJP, Sanders AD, Zhao X, Malhotra A, Porubsky D, Rausch T, et al. Multi-platform discovery of haplotype-resolved structural variation in human genomes. Nat Commun. 2019;10(1):1784.

3. Miga KH, Newton Y, Jain M, Altemose N, Willard HF, Kent WJ. Centromere reference models for human chromosomes X and Y satellite arrays. Genome Res. 2014;24(4):697-707.

4. Garrison E, Marth G. Haplotype-based variant detection from short-read sequencing. arXiv 1207.3907 [Preprint]. 2012 [cited 2020 Dec 8]. Available from: https://arxiv.org/abs/1207.3907

5. Porubsky D, Garg S, Sanders AD, Korbel JO, Guryev V, Lansdorp PM, et al. Dense and accurate whole-chromosome haplotyping of individual genomes. Nat Commun. 2017;8(1):1293.

6. Kent WJ, Sugnet CW, Furey TS, Roskin KM, Pringle TH, Zahler AM, et al. The human genome browser at UCSC. Genome Res. 2002;12(6):996-1006.
