# Supplementary figures and images for: InvertypeR: Bayesian inversion genotyping with Strand-seq data
Source: BMC Genomics. 2021 Jul 31;22:582. doi: 10.1186/s12864-021-07892-9 (PMC8325862; doi:10.1186/s12864-021-07892-9)

HG00512  
GRCh38

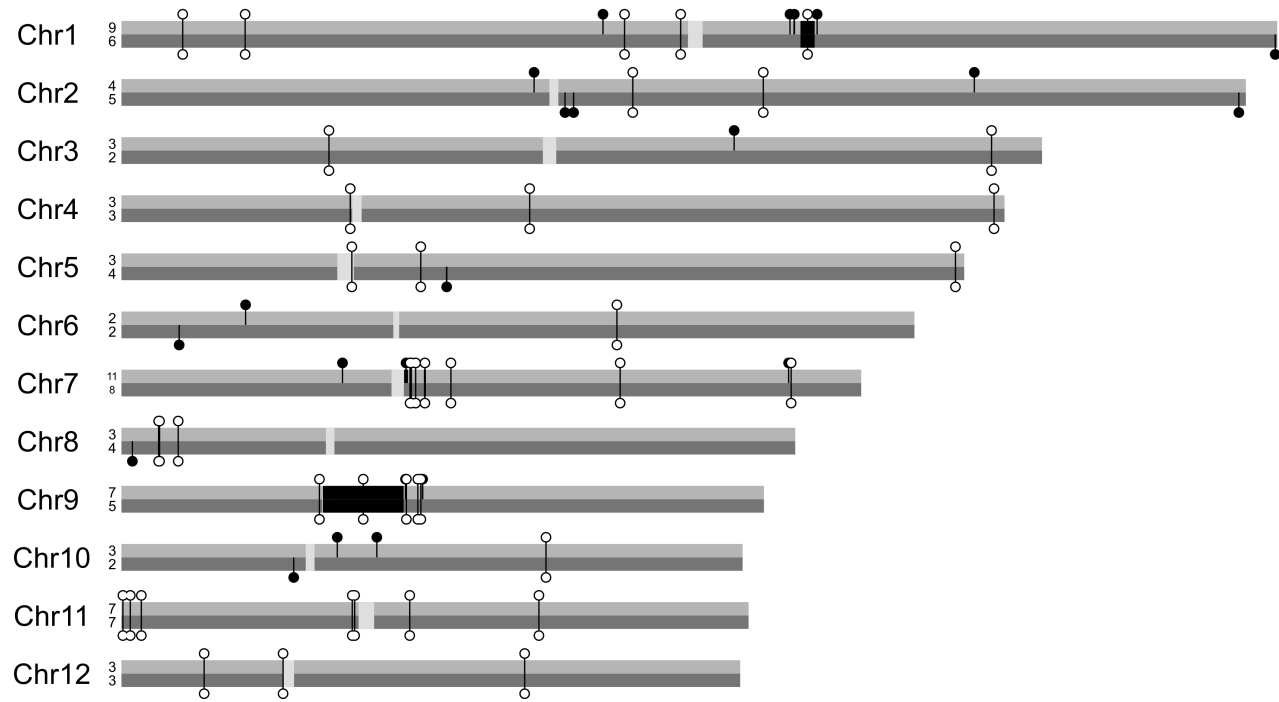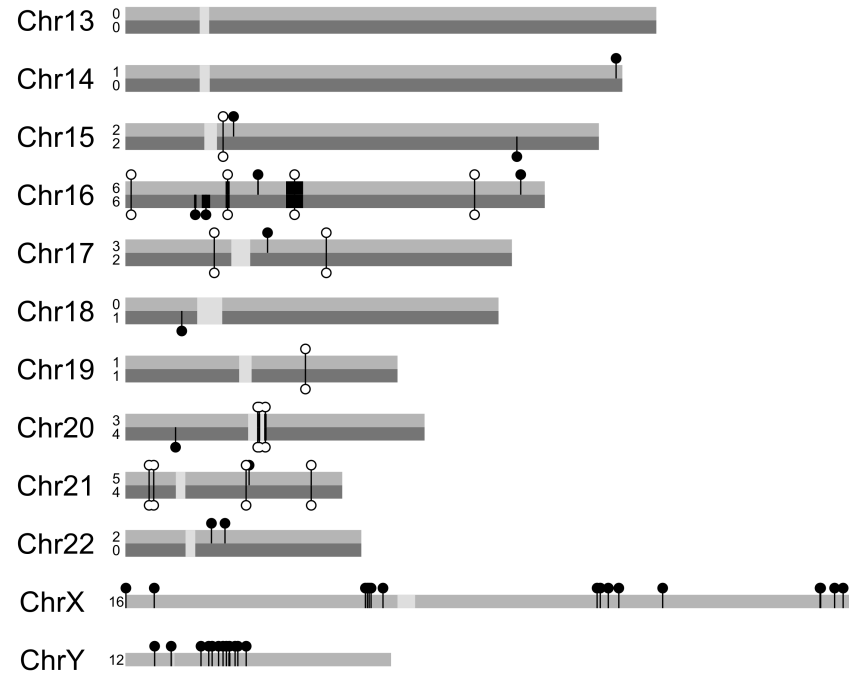

Supplement: Supplementary file 2 — Additional file 2: Supplemental Figure S2. [file 12864_2021_7892_MOESM2_ESM.pdf]

HG00513  
GRCh38

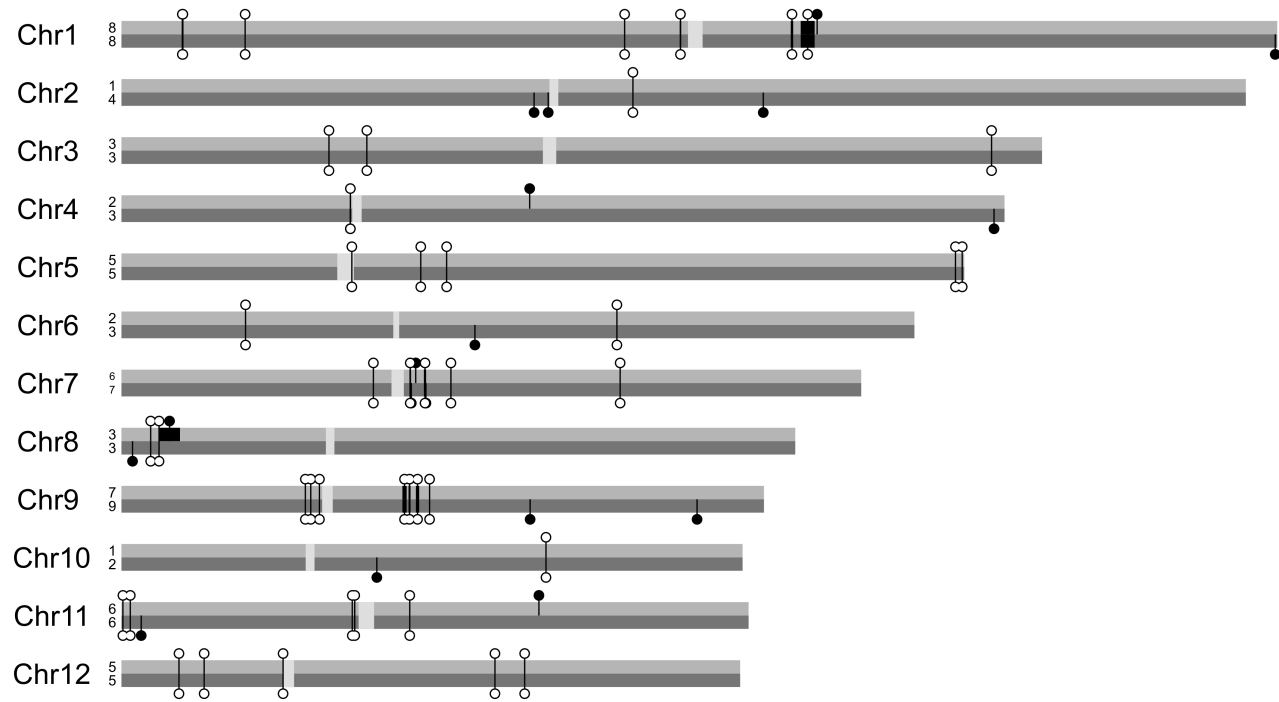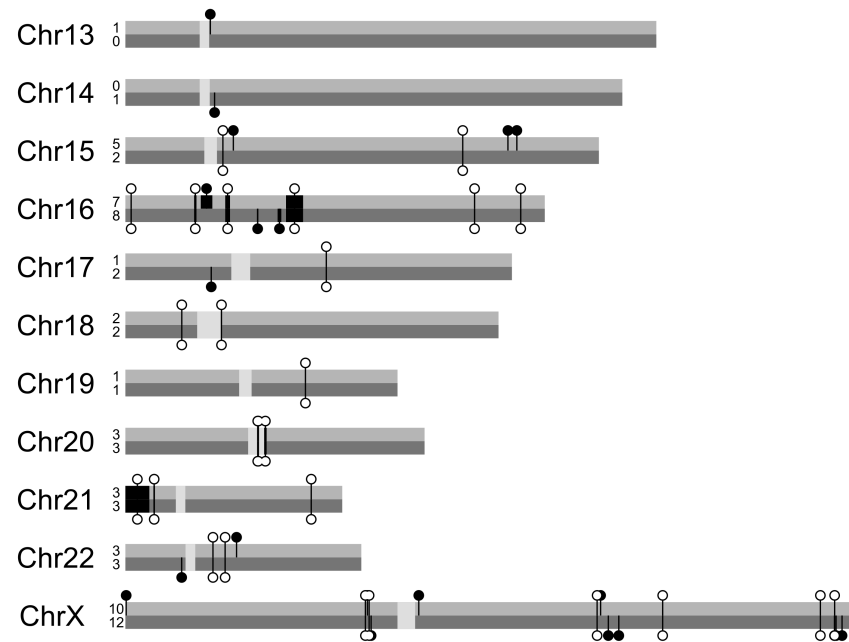

Supplement: Supplementary file 3 — Additional file 3: Supplemental Figure S3. [file 12864_2021_7892_MOESM3_ESM.pdf]

HG00514  
GRCh38

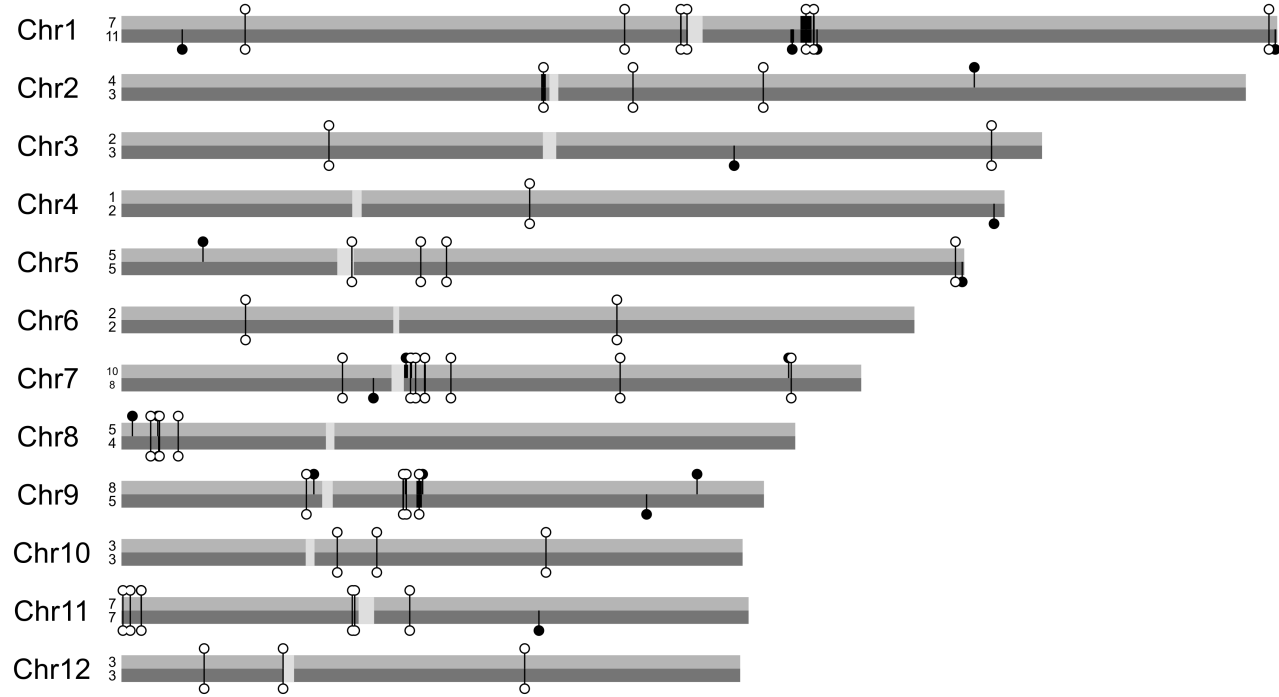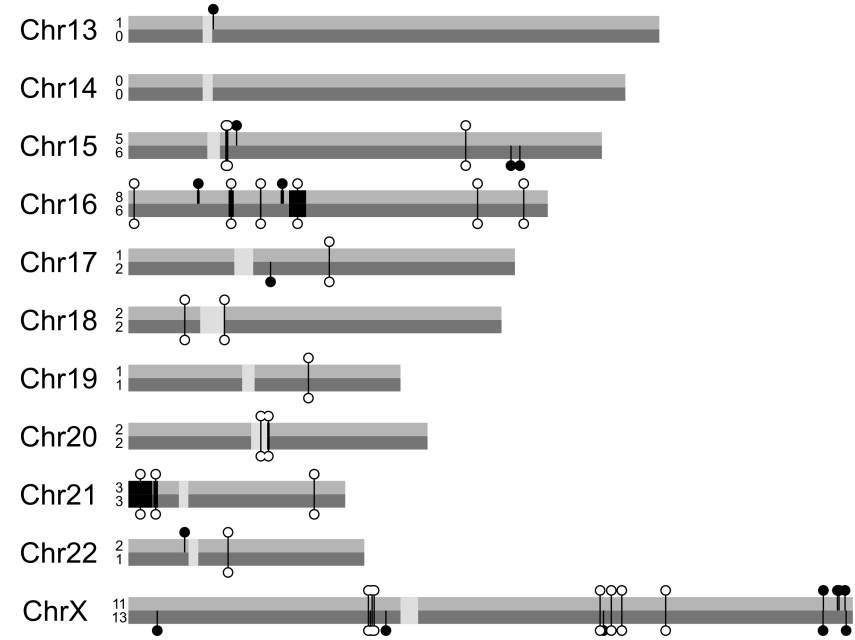

Supplement: Supplementary file 4 — Additional file 4: Supplemental Figure S4. [file 12864_2021_7892_MOESM4_ESM.pdf]

HG00731

GRCh38

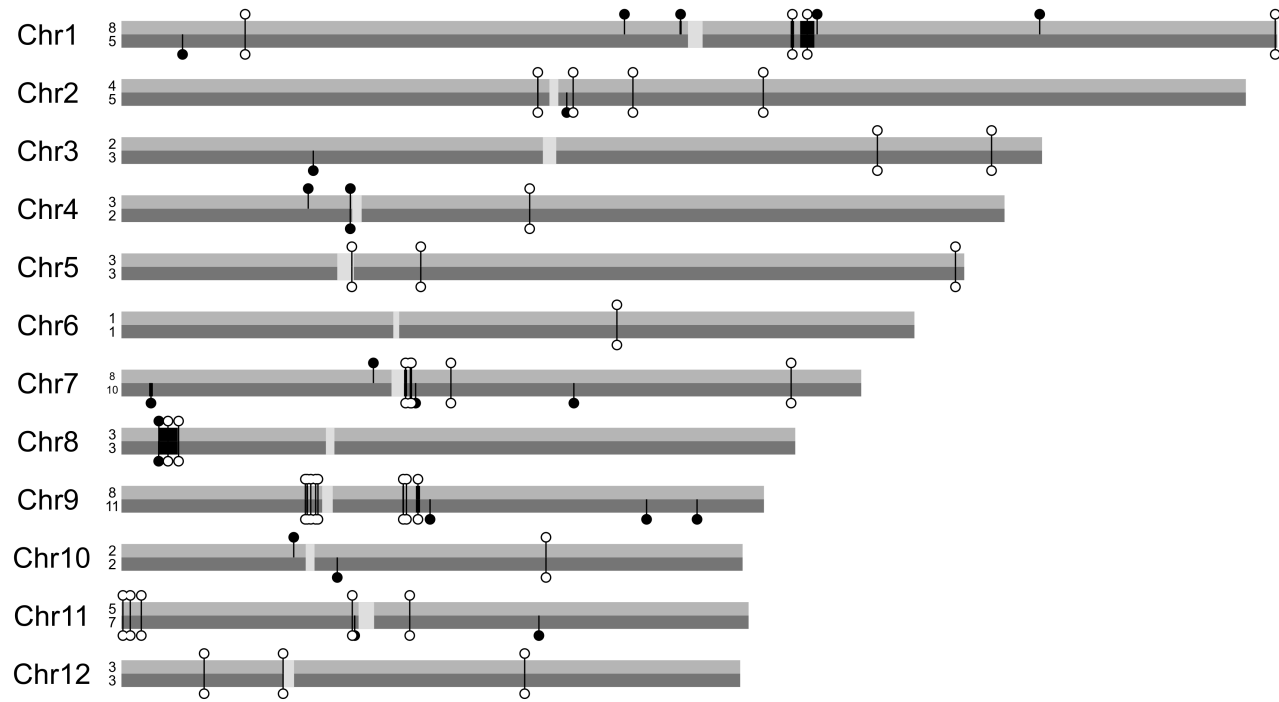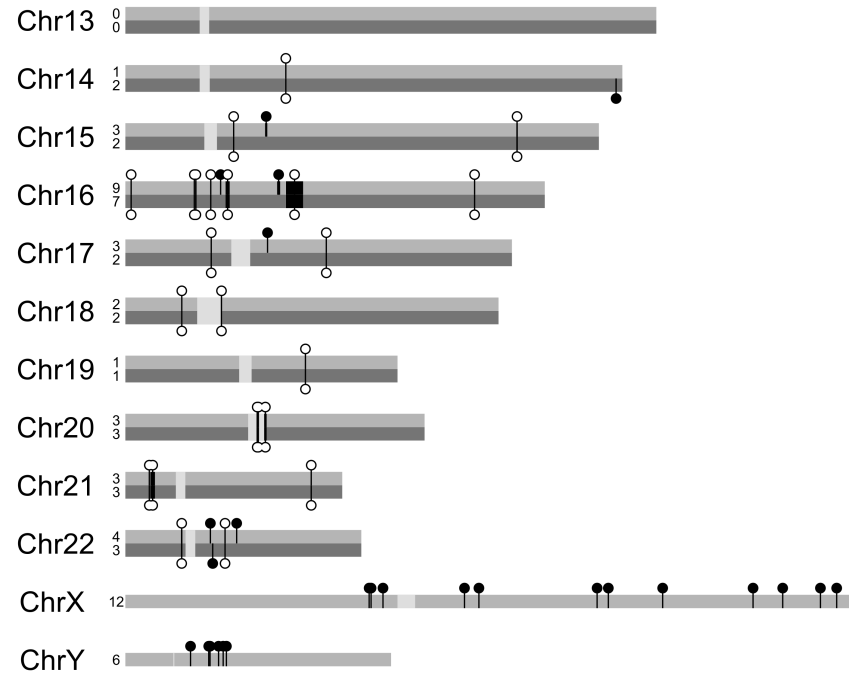

Supplement: Supplementary file 5 — Additional file 5: Supplemental Figure S5. [file 12864_2021_7892_MOESM5_ESM.pdf]

HG00732

GRCh38

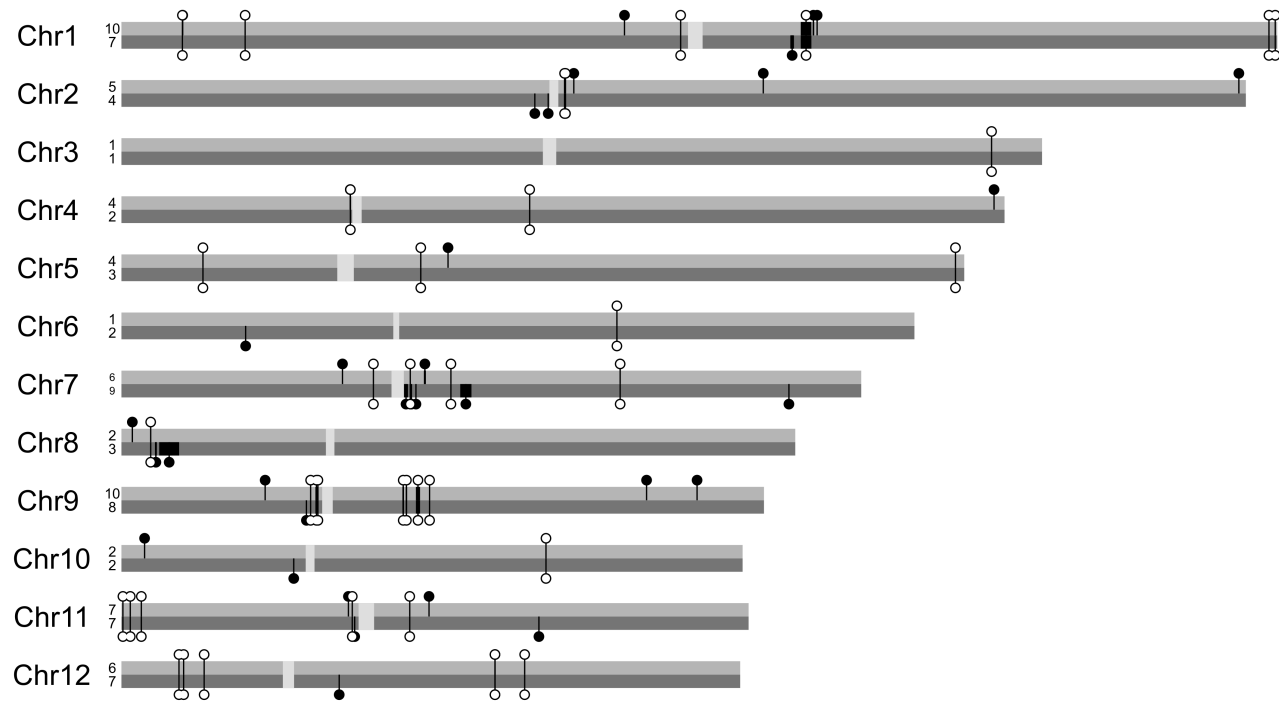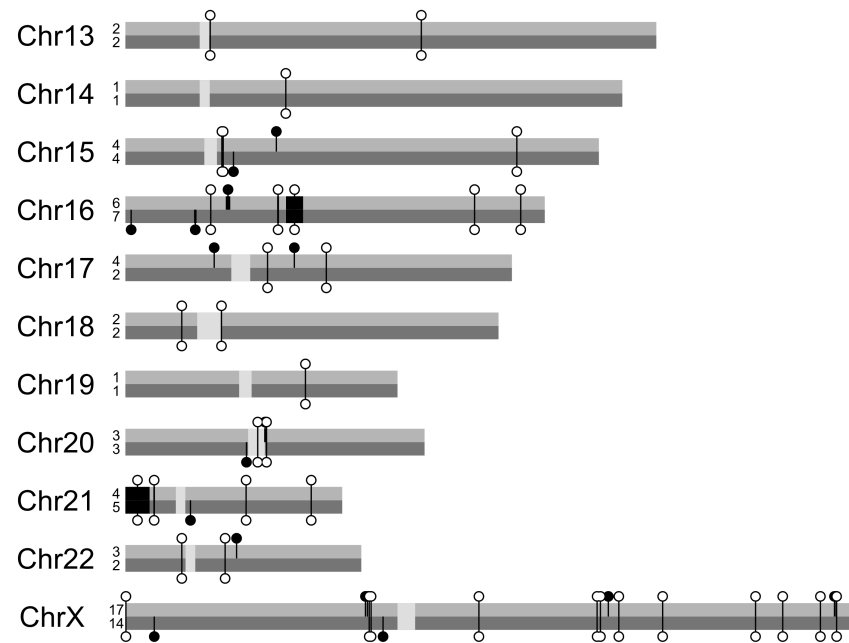

Supplement: Supplementary file 6 — Additional file 6: Supplemental Figure S6.. [file 12864_2021_7892_MOESM6_ESM.pdf]

HG00733  
GRCh38

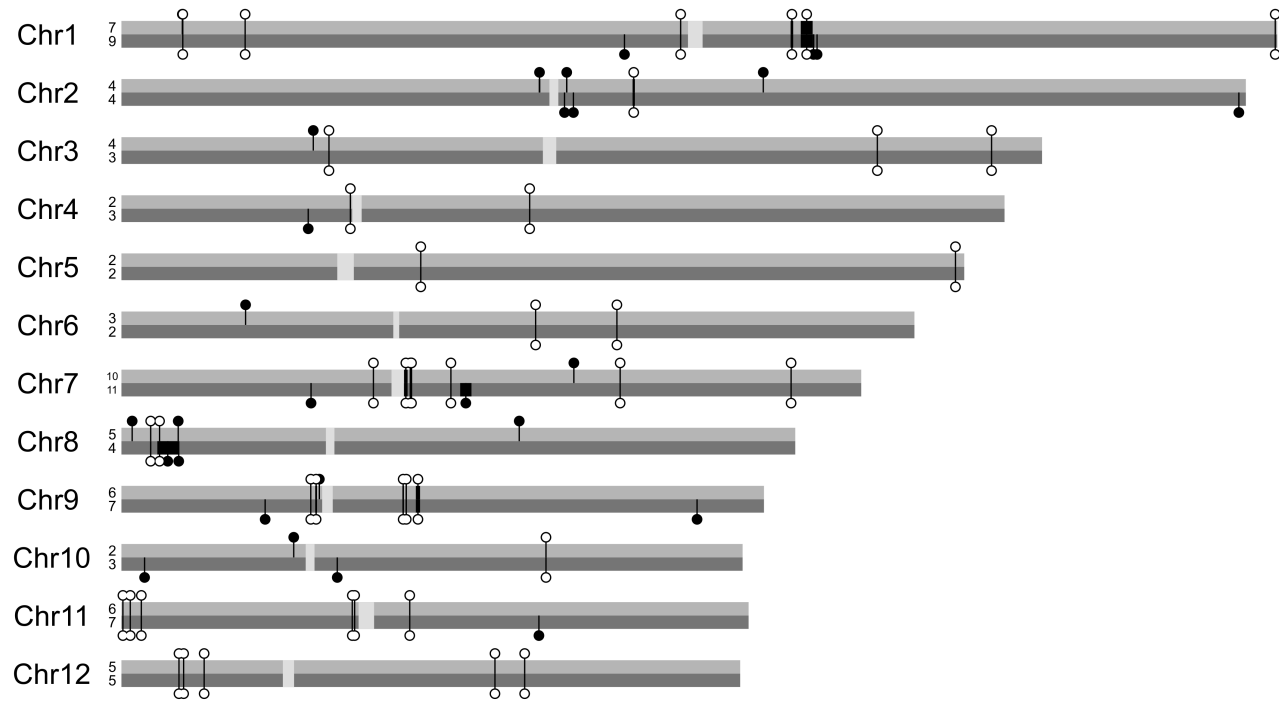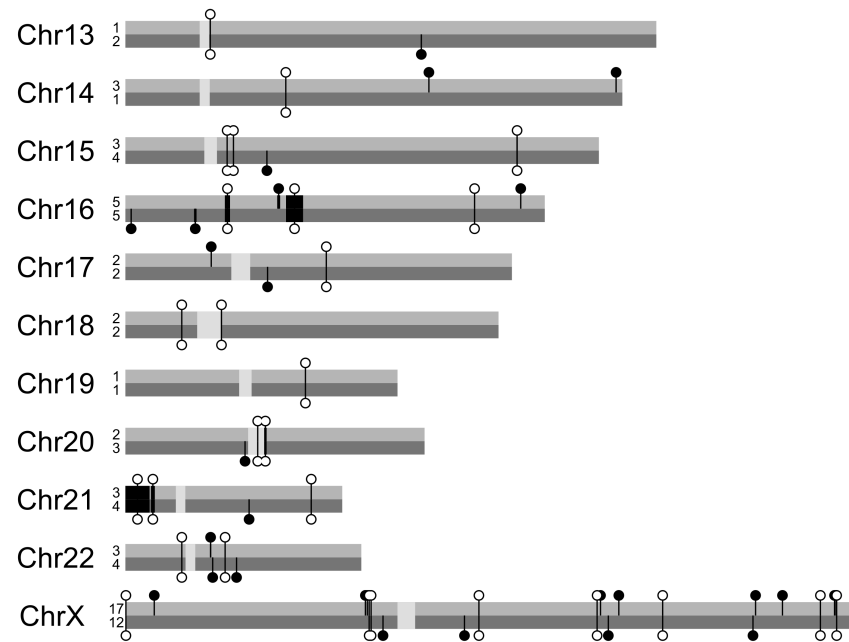

Supplement: Supplementary file 7 — Additional file 7: Supplemental Figure S7. [file 12864_2021_7892_MOESM7_ESM.pdf]

NA19239  
GRCh38

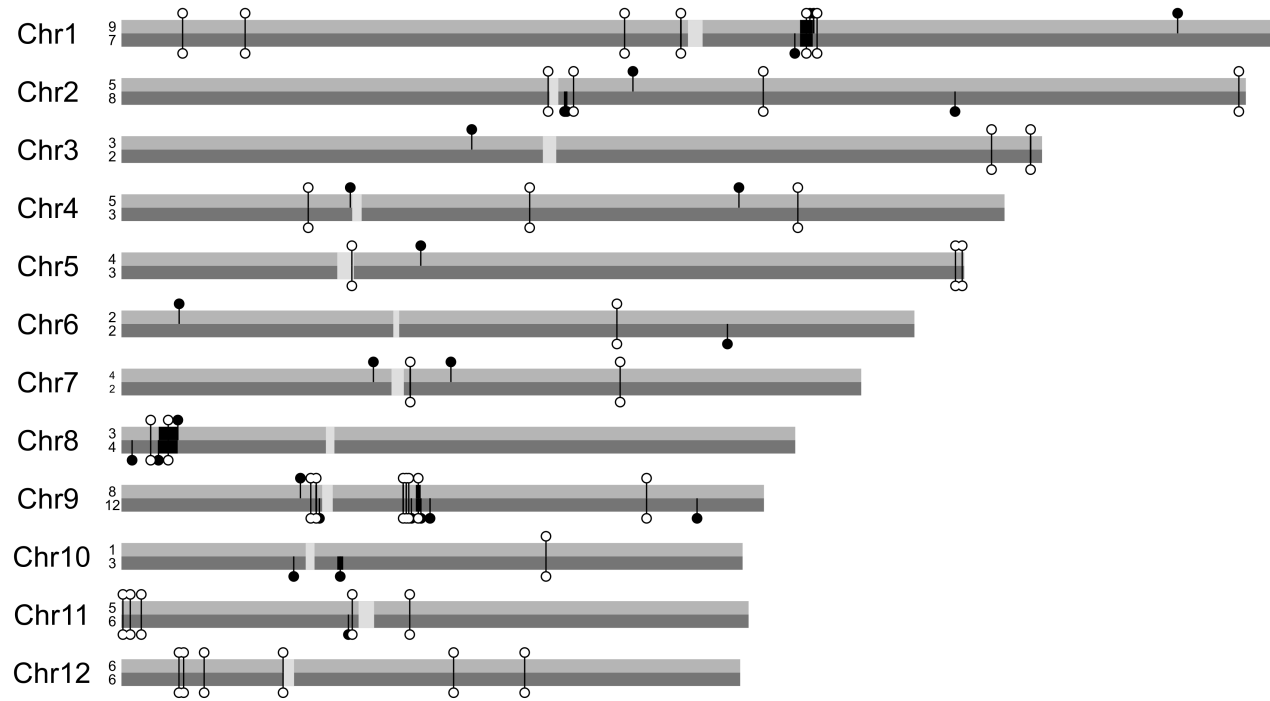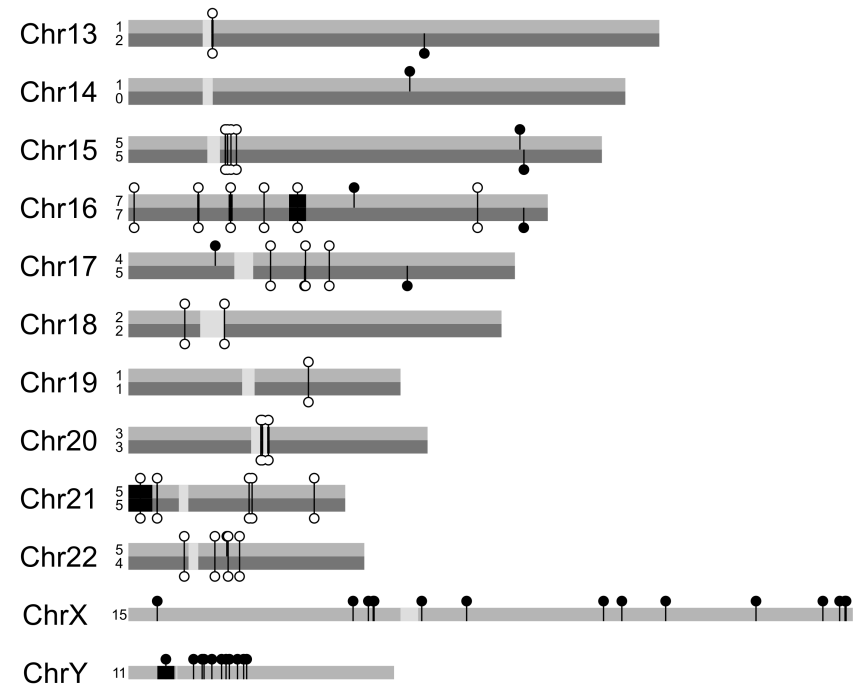

Supplement: Supplementary file 8 — Additional file 8: Supplemental Figure S8. [file 12864_2021_7892_MOESM8_ESM.pdf]

NA19238  
GRCh38

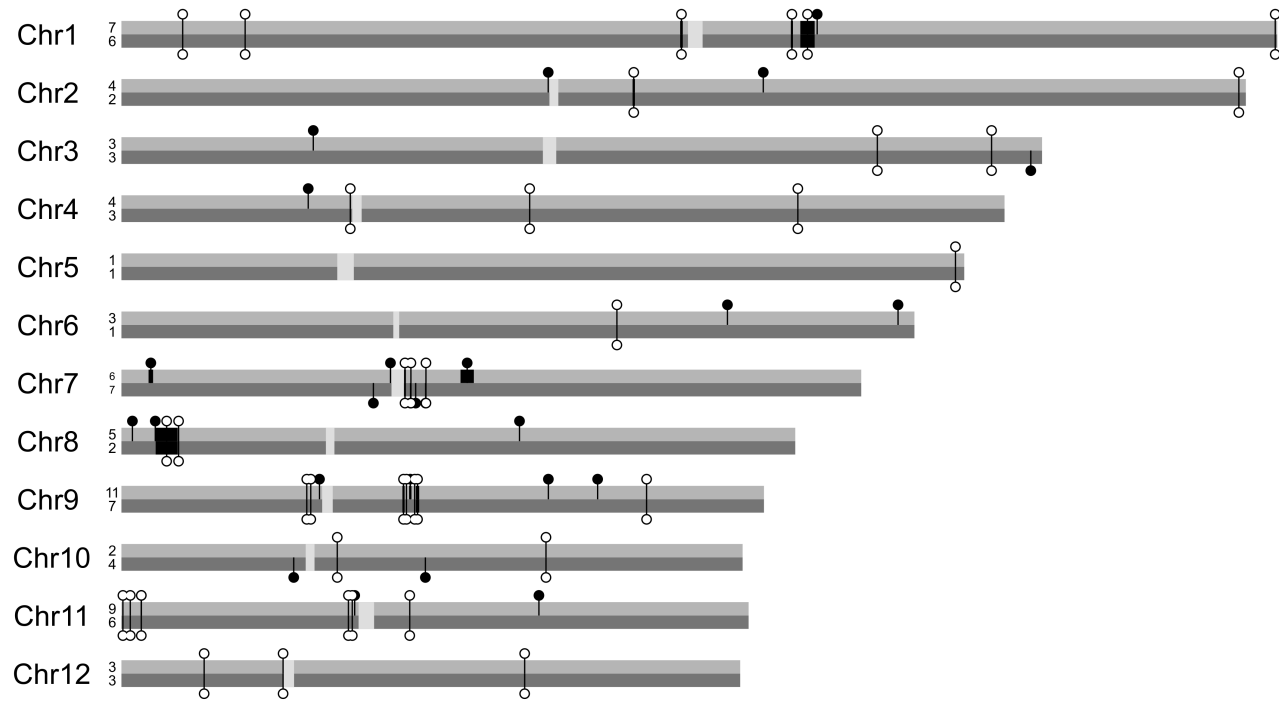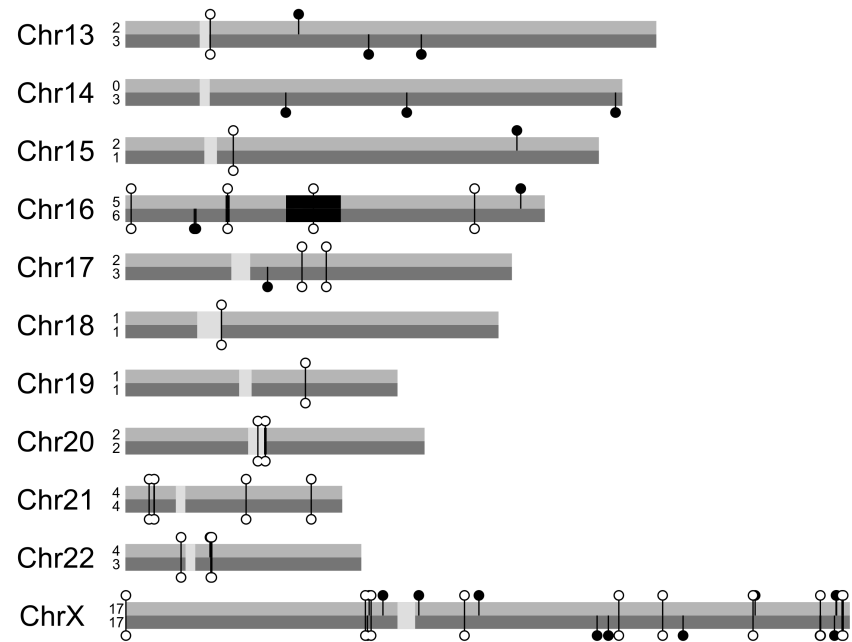

Supplement: Supplementary file 9 — Additional file 9: Supplemental Figure S9. [file 12864_2021_7892_MOESM9_ESM.pdf]

NA19240  
GRCh38

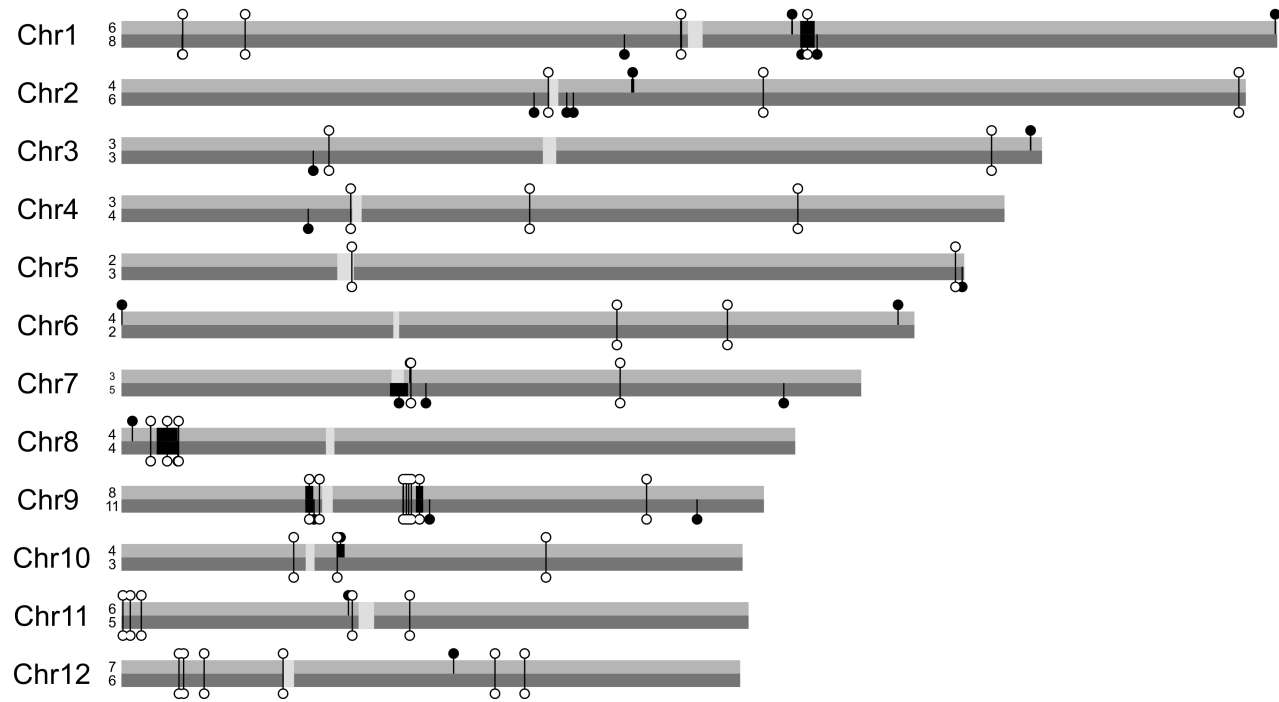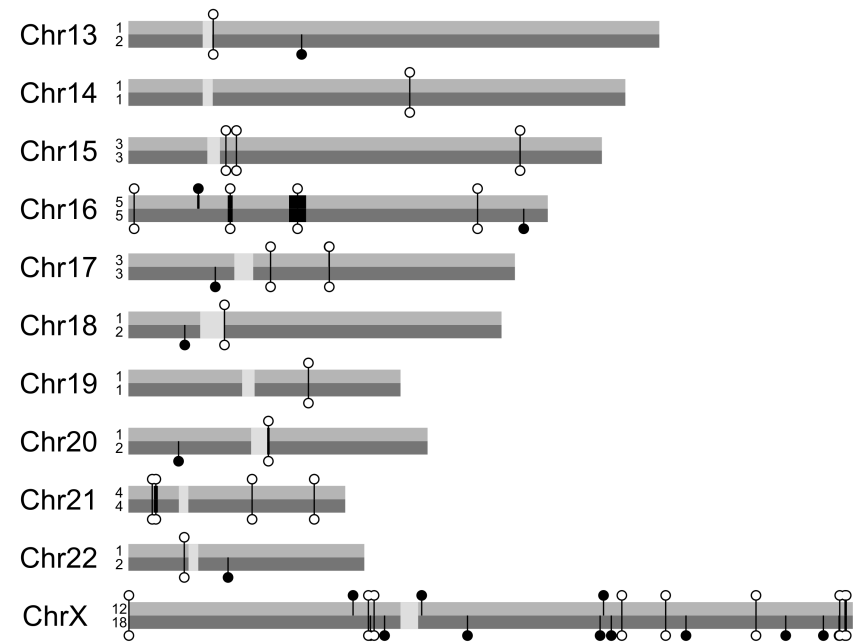

Supplement: Supplementary file 10 — Additional file 10: Supplemental Figure S10. [file 12864_2021_7892_MOESM10_ESM.pdf]
